# Anæmia

**Published:** 1891-05-02

**Authors:** 


### THE PRACTITIONER'S MIRROR AND RETROSPECT.

[*The Editor will be glad to receive offers of co-operation and contributions from members of the profession. All letters should be addressed to* THE EDITOR, THE LODGE, PORCHESTER SQUARE, LONDON, W.]

### MEDICINE.

### ANÆMIA.

The pathology of the various forms of anæmia is so interwoven with the physiology of the blood and its production that it seems impossible to come to any useful results till the latter subject has been placed within the domain of accurate knowledge.

Dr. Stephen Mackenzie has recently given an able survey of both fields for research in his Gulstonian lectures. He considers that all the evidence tends to show that the red corpuscles are produced in the venules of the marrow, and to a smaller extent, if at all, in the glandular organs. Whether they are multiplied by division, or produced from marrow cells, or from white corpuscles, is still debated. Edington has traced them in every stage of growth from small colourless cells, which again he has succeeded in photographing at the moment of extrusion from a mother cell. A constant process of formation, however, is going on, for the average life of each corpuscle is limited to about a fortnight, when it becomes disintegrated either in the vessels of the marrow or in those of the digestive organs, its colouring matter being either handed over to the bile and other secretions, or used over again in new corpuscles.

According to Dr. William Hunter this destruction is of two kinds, a slow passive decay and an active destruction. The former occurs more especially during fasting and in later life; the latter process is more marked in young animals when nutrition is active, and especially during digestion. In the latter, too, the pigment is excreted by the liver as bile pigment, the formation of which is thus "entirely subserved by the active destruction of blood," and which forms an index of the amount so destroyed. Though there is strong probability in this view, we agree with Dr. Mackenzie that the evidence is inadequate to prove that the bile pigments are derived solely from the blood, and that they are a measure of the active destruction. Why should the liver cells be less competent to manufacture their pigment than the original corpuscles were? One thing is clear, however. There is a constant production and removal of the corpuscles of the blood, and yet in health there is a balance maintained between this creation and destruction. Their numbers approximate to a certain normal point, much as the temperature is kept at an equilibrium between heat production and heat loss.

Every kind of anæmia depends on a failure to maintain this balance of normal functional red corpuscles. The corpuscles may be deficient in number; sometimes it is that they are normal in number but deficient in quality, especially in hæmoglobin, or few in number and accompanied by an excess of white corpuscles, or deficient both in number and quality together. By tracing the origin of this deficiency, the various forms of anæmia can be classified according as they arise from disordered hæmatogenesis or disordered hæmatolysis. Thus, in chlorosis, leucocythemia, Hodgkin's disease, and anæmia splenica, we have probably instances of a failure of formation, while in traumatic anæmia, hæmatinuria, and pernicious anæmia we have, according to recent observers, excessive corpuscle destruction.

Now it is a curious fact that an excess of corpuscles is comparatively rare if not unknown. "We do not find," says Fagge, "the number of corpuscles or the amount of hæmoglobin increased in any form of disease." Plethora has disappeared from the text-books, whether produced by an excess of hæmatogenesis or from deficiency of hæmatolysis. Here, again, we are reminded of the heat regulating function. How rarely do we meet with subnormal as compared with pyrexial temperatures, and how much less is their range. A rise of 10 degrees may occur, but a fall of 10 degrees is practically unknown. In the formation of blood corpuscles, as in that of the hypothetical thermogen, we have to do with an inhibitory anabolic process—one, that is, of building up, while in the destruction of corpuscles, as in that of the evolution of heat, there is a katabolic process, that is one dissipative of energy. Now, though these two processes are closely connected together and inter-dependent, the constructive inhibitory process is more delicate, and fails usually on the side of defect, while the katabolic easily runs into excess. Or we may say the greater the amount of material stored up during inhibition the stronger becomes the tendency towards the liberation of force. If we accept Dr. William Hunter's views on the destruction of blood corpuscles, we may express this tendency in a particular way. He believes, as we have seen, that the active destruction of corpuscles goes on in the liver and intestinal mucosa most rapidly during digestion, and sinks to a minimum during starvation. So that whenever increased nutrition gives material for building up new corpuscles, the very presence of this food increases the activity of the spleen and intestinal tissues, and increased destruction comes into play. While conversely, even if nutrition ceases entirely, passive, and a little active, destruction still goes on. Whatever may be the true explanation, the rarity of an excess of normal corpuscles and frequency of diseases where they are deficient is noteworthy.

Again, if Dr. Hunter's views are correct, it will be important to discover whether in cases where production and destruction are both rapid, the organism is ever unable to cope with the excess of corpuscles thrown out. Are there, for example, any diseases of the liver produced by failure to excrete the excess, or diseases of it followed by insufficient or too great excretion?

The changes in the corpuscles must at times be very rapid. Dr. Copeman has proved that in hæmatinuria 800,000 per cubic millimetre have been destroyed in a quarter of an hour, and yet that nearly that number were replaced before the end of a week. In this disease the corpuscles, indeed, are destroyed in the general circulation, and the hæmoglobin excreted as such by the kidneys, while in pernicious anæmia, Dr. Hunter holds that the destruction occurs in the portal system, and all the hæmoglobin is changed into biliary and urinary pigments. In the latter affection the corpuscles sink to about 15 per cent. before death, but the percentage of hæmoglobin in each is greater than the normal. The urine is in many cases of darker colour than usual, and contains a greater quantity of iron, which Hunter lays stress on in support of his view that active destruction takes place in the portal system. However, the colour is not invariably present. From the presence of certain aromatic sulphates and ptomaines in the urine, he argues for the action of septic organisms as an essential factor in this disease. These are absorbed from the intestinal tract and affect the blood in the portal vessels. The evidence is, indeed, by no means complete, but there is a good deal which confirms it in the febrile condition and toxic nervous symptoms. At present we have not learnt that very successful results have followed the administration of disinfectants, such as beta naphthol, which he recommends.

The administration of arsenic does certainly check for a time the progress of the disease. We do not know whether this is due to its antiseptic action, or to its influence on the liver cells, or to its direct preservative action on the corpuscles, whose break up it prevents even when added to them outside the body.

For the forms of anæmia depending on failure of hæmatogenesis, we find advocates of every form of iron, but as all iron seems to be reduced to a chloride or albuminate in the stomach, the relative interference of different preparations with the digestion is what we have chiefly to think of. Even the pill forms cannot, it appears, claim the minor advantage of not affecting the teeth. Indeed, the great thing is to increase the digestive process, for sufficient iron is, after all, present in the food itself, if we can abstract it. This consideration is strengthened by the beneficial effects we see in the use of aperients. Certainly assimilation is improved by them, whether they act directly on the digestive organs, or, as has been suggested, by clearing away organisms which interfere with their activity. It must not be thought, however, that iron has no specific action. It certainly is absorbed in large quantities, though a proportion passes away in the fæces, and the improvement which follows its careful administration is so frequently seen and so decided that we cannot doubt of its being due to the drug. Unhappily the effects are not permanent, and the defective assimilation of iron is apt to recur both after recovery from chlorosis and after some progress seems to have been made in stopping the progress of the more malignant forms of anæmia.

